# Low-Dose Subarachnoid Anesthesia Combined with PENG and FLCN Blocks Reduces Hypotensive Episodes Without Compromising Anesthetic Depth and Duration in Hip Fracture Surgery: A Retrospective Observational Study

**DOI:** 10.3390/medicina61101808

**Published:** 2025-10-09

**Authors:** Daniel Salgado-García, Agustín Díaz-Álvarez, José L. González-Rodríguez, María R. López-Iglesias, Eduardo Sánchez-López, Manuel J. Sánchez-Ledesma, María I. Martínez-Trufero

**Affiliations:** 1 Department of Anesthesiology and Critical Care, Salamanca University Hospital, Gerencia Regional de Salud de Castilla y León (SACYL), Paseo de la Transición Española, 37007 Salamanca, Spain; daniel.salgado@salud.madrid.org (D.S.-G.); jlgonzalezr@saludcastillayleon.es (J.L.G.-R.); mrlopezig@saludcastillayleon.es (M.R.L.-I.); esanchezl@saludcastillayleon.es (E.S.-L.); mjsanchezl@saludcastillayleon.es (M.J.S.-L.); imtrufero@saludcastillayleon.es (M.I.M.-T.); 2 Department of Surgery, Faculty of Medicine, Salamanca University, Calle Alfonso X el Sabio, 37007 Salamanca, Spain; 3 Instituto de Investigación Biomédica de Salamanca de la FIESCYL (IBSAL-FIESCYL), Paseo de San Vicente 182, 37007 Salamanca, Spain

**Keywords:** elderly, frail, fractures, hip, hypotension, hemodynamics, anesthesia, spinal, block, nerve

## Abstract

*Background and Objectives*: In the context of hip fracture surgeries, episodes of hypotension are common, and have been associated in various studies with increased complications and mortality. The latest clinical guidelines recommend close hemodynamic management. Our research team hypothesized that the use of peripheral nerve blocks in this surgery could help adjust the doses of subarachnoid anesthesia for these procedures, thereby limiting the hypotensive episodes, without compromising an adequate depth and duration of intraoperative anesthesia. *Materials and Methods*: A retrospective study of 184 elderly patients undergoing hip fracture surgery is proposed. In total, 76 patients were operated under subarachnoid anesthesia using 9.5 mg of hyperbaric bupivacaine 0.5% and 10 mcg of fentanyl (Group S), while 108 received a reduced dose of 5 mg hyperbaric bupivacaine 0.5% and 10 mcg of fentanyl, supplemented by preoperative PENG and FLCN blocks (Group B). The main outcome of this study is to compare the number and duration of hypotensive episodes, and its secondary outcome is to compare the use of vasoactive drugs between the groups. *Results*: The number of hypotensive episodes and their duration were lower in Group B: −12.94 min (−8.57 to −18.03, *p* = 0.000). The consumption of vasoactive drugs did not reach statistical significance. None of the patients in Group B required supplementary intraoperative anesthesia. *Conclusions*: Reducing the dose in subarachnoid anesthesia is associated with better hemodynamic control in hip fracture surgeries, and PENG + NFCL blocks are proposed as an appropriate adjunct to ensure adequate anesthetic depth and duration despite a substantial subarachnoid anesthesia dose adjustment.

## 1. Introduction

The association of intraoperative hypotension episodes with increased mortality and morbidity in non-cardiac surgery is an established and well-documented fact [1]. In the case of hip fracture surgeries, hypotensive episodes are common and have been more frequently associated with general anesthesia [2]. The literature contains various previous studies advocating an increase in the occurrence of complications (such as delirium, stroke, arrhythmias, myocardial infarction, or renal failure) [3,4], and mortality [5] associated with hypotensive events in the context of hip fractures. Some of these studies also demonstrate a relationship between the doses of anesthetic used and the occurrence of hypotensive events [2,6], which, in turn, has an effect on the clinical outcomes measured in these studies.

Based on these results, the latest clinical guideline on hip fracture surgery [7] emphasizes the importance of careful hemodynamic management during the procedure, advocating for the adjustment of anesthetic doses to the patient’s clinical context. This is particularly relevant given that the typical profile for these surgeries involves elderly patients [8,9], who usually present with frailty [10], multiple comorbidities, and polypharmacy [11,12,13]. Furthermore, the clinical guideline highlights the routine use of peripheral nerve blocks in the perioperative period of these interventions [7], such as femoral nerve block, fascia iliaca block, and Pericapsular Nerve Group (PENG) block.

Considering these recommendations, our intention was to explore addressing both of them at a time, from the perspective of performing peripheral blocks, in this case, the Peripheral Nerve Group (PENG) and Femoral Lateral Cutaneous Nerve (FLCN) blocks. Thus, beyond the already well-documented effectiveness of this blocks in providing analgesia and its associated benefits, we aimed to explore whether they could also contribute to improve intraoperative hemodynamic management of patients. In this regard, we hypothesize that the PENG and FLCN blocks could assist in hemodynamic control by allowing for a reduction in the doses of subarachnoid anesthesia (which would evidently lower the incidence of hypotension) but without compromising the adequacy of intraoperative anesthesia, owing to the additional analgesic control that these techniques provide.

## 2. Materials and Methods

### 2.1. Study Design and Ethics

To study this hypothesis, we proposed a single-center, retrospective, observational study. Prior to conducting this study, approval for its implementation was obtained from the Ethics Committee for Drug Research of the Health Area of Salamanca (CEIm PI 2021 11 893, dated 22 November 2021) in accordance with the International Council for Harmonization (ICH) guidelines for Good Clinical Practice (GCP). As this is a retrospective study, informed consent was not directly obtained from the patients involved. However, the informed consent provided to patients at our institution prior to their intervention includes a clause by which they give their specific consent for the anonymized use of health data derived from their medical care for studies related to their clinical process.

### 2.2. Group Characteristics and Data Collection

Data collection for this study was conducted through a review of intraoperative anesthesia records from the patients’ paper medical records, who underwent hip fracture surgery during 2022 at our institution, facilitated by the Department of Anesthesiology and Resuscitation at our center. The inclusion criteria for this study comprised patients aged over 65 with hip fractures who were surgically treated at our institution. No exclusions were made because, being a retrospective observational study, we only included in our sample those patients who met the inclusion criteria by reviewing the database of our institution. For this reason, there were no cases of dropout, as we ensured that all included patients did not present any extraordinary factors during their intraoperative course that could have been potentially limiting. For this study, the analyzed patients were divided into two groups according to the type of intraoperative anesthetic management they received. The control group consisted of patients who exclusively received subarachnoid anesthesia, comprising 9.5 mg of hyperbaric bupivacaine 0.5% (Braun, Melsungen, Germany) and 10 mcg of fentanyl (Kern, Madrid, Spain), contained in a 2 mL solution administered. This anesthetic management reflects the clinical practice at our center in cases where the PENG block is not performed prior to subarachnoid anesthesia, due to rejection of the technique, contraindications for its implementation, or the urgency of the surgical procedure. In the case group, patients were included who received a reduced dose of 5 mg hyperbaric bupivacaine 0.5% and 10 mcg of fentanyl, supplemented by preoperative PENG and Femoral Lateral Cutaneous Nerve (FLCN) blocks. These patients reflect the usual anesthetic clinical practice in our center for hip fracture interventions, given the strong recommendation in clinical guidelines for hip fracture regarding the benefits of peripheral nerve blocks in this context. Among the available options, the PENG block has demonstrated efficacy as a technique for perioperative pain management, while reducing the incidence of motor blockade of the lower limb compared with femoral nerve block or fascia iliaca block.

These blocks were performed in the operating room at the patient’s bedside in a supine position, prior to their transfer to the operating table and after EKG, NIBP, and pulse oximetry monitoring. The blocks were performed sterilely and ultrasound-guided using a convex probe and an 80 mm needle (Braun, Germany) for the PENG block, and a linear probe and a 50 mm needle (Braun, Germany) for the FLCN block. For this, 20 mL of local anesthetic was administered for the PENG block and 5 mL for the FLCN in a single dose, using either levobupivacaine 0.25% (Abbvie, North Chicago, IL, USA) or ropivacaine 0.375% (Fresenius Kabi, Bad Homburg, Germany). After confirming the analgesic effect of the block, the patients were transferred to the surgical table to receive subarachnoid anesthesia comprising 5 mg of hyperbaric bupivacaine 0.5% (Braun, Germany) and 10 mcg of fentanyl (Kern, Spain), contained in a 1 mL solution administered. Specifically, this case group was obtained through the sample included in the PENG-CAD clinical trial, conducted by this same research team prior to the present study, which was registered and is available at ClinicalTrials.gov under reference NCT04773301 [14]. The FLCN block is performed at our institution as part of multimodal analgesia to cover incisional pain, which differs from the pain originating from the hip fracture and its fixation that the PENG block covers, thus having no influence on the target variables of this study.

In both groups, subarachnoid anesthesia was performed after the establishment of a peripheral venous line, patient monitoring with EKG, NIBP, and pulse oximetry, and the initiation of 2–3 L min-1 oxygen and sedation with titrated midazolam boluses or continuous propofol infusion, as appropriate. The procedure was performed sterilely, with the patient in a sitting position on the surgical table, at the L2–L3 or L3–L4 level using 25 G pencil-point subarachnoid needles, after confirming cerebrosubarachnoid fluid reflux. Vasoactive drugs were administered in cases where mean arterial pressure (MAP) was below 60 mmHg, using ephedrine or phenylephrine based on the patient’s heart rate levels (ephedrine if low, phenylephrine if high). If blood pressure levels were not controlled after 5 min (the necessary time for these drugs to counteract hypotension), subsequent doses of the most appropriate drug was administered depending on the patient’s heart rate, to balance the opposing effects of ephedrine and phenylephrine on cardiac receptors, aiming to maintain adequate heart rate levels. After surgery, which was approached in a lateral decubitus position, the patients were transferred to the post-anesthesia care unit, and later to the Traumatology hospitalization ward when their clinical condition allowed.

### 2.3. Study Objectives

The primary objective of this study is to compare the number and duration of intraoperative hypotensive episodes between both groups, with the hypothesis that both will be fewer in the group of patients receiving the PENG block, which would allow for the use of lower doses of subarachnoid anesthesia without compromising adequate surgical anesthesia depth and duration.

As a secondary objective, we aim to compare the intraoperative use of vasoactive drugs between the study groups, with the hypothesis that it will be higher in the group exposed only to subarachnoid anesthesia, given the higher dose of local anesthetic used in this group.

### 2.4. Variables, Data Handling, and Statistical Analysis

To achieve the proposed objectives, the research team collected the following data from the examined medical records: Demographic data (Age, Sex, Identification code), Type of fracture (Subcapital, Pertrochanteric, or Subtrochanteric), Type of surgical intervention (Intramedullary nailing or hip arthroplasty), Duration of surgery (minutes), Hypotension (MAP < 65 mmHg, duration in minutes and number of episodes), Use of vasoconstrictors (ephedrine (mg) and phenylephrine (mg)), Type of anesthesia (subarachnoid anesthesia, subarachnoid anesthesia + PENG-FLCN blocks).

In Spain, the Organic Law 3/2018 of December 5, complementing Regulation (EU) 2016/679 of the European Parliament and of the Council of 27 April 2016, mandates the implementation of high-level security measures for handling health data. In compliance with these requirements, study data was protected by identifying patients by a code, and only the research team was allowed to review the patients’ medical records. The code used did not permit the identification of subjects and did neither include nor collect identifying information.

A descriptive analysis plan for the collected variables was approved by the authors before the analysis began. Non-categorical variables are expressed as median (interquartile range—IQR). Categorical variables are expressed as percentages or frequencies. After conducting appropriate normality tests (Kolmogorov–Smirnov), differences in the samples were determined using frequency analysis, mean comparison, Student’s *t*-test or Mann–Whitney U test depending on the results of the normality tests, and Chi-square (χ^2^).

## 3. Results

After reviewing the medical records of patients over 65 years of age who underwent hip fracture surgery at our institution during the year 2022, 76 patients were selected for the control group (subarachnoid anesthesia—group S) and 108 patients for the case group (subarachnoid anesthesia plus blocks—group B). The demographic characteristics of the study sample are shown in Table 1. There are no significant differences between the two groups, except for the duration of the intervention, in which a statistically significant difference was found, being shorter in Group B.

**Figure 1:**
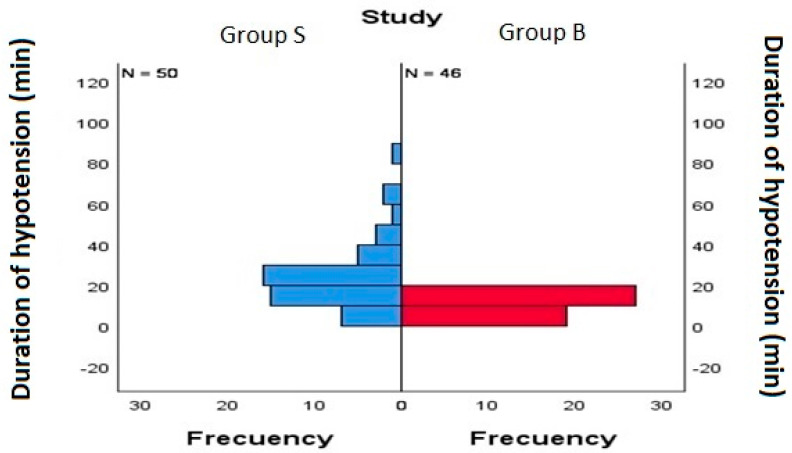
Distribution of duration of hypotension between groups. Group S (subarachnoid anesthesia), Group B (subarachnoid anesthesia + blocks).

The duration of hypotension (MAP < 65 mmHg) in this study has been reflected in intervals of 5 min, corresponding to 1 episode. Table 2 shows the median number of hypotension episodes, and their median duration, in each of the study groups and, therefore, in relation to the type of anesthesia used. Data from Table 2 is displayed visually in Figure 1. Table 3 displays the median amount of vasoconstrictors used in each group.

## 4. Discussion

In this article, we attempt to present our findings on the combination of PENG and FLCN blocks with low-dose subarachnoid anesthesia for hip fracture surgery in elderly patients, regarding the hypotension events and the intraoperative anesthetic adequacy.

The demographic characteristics of the sample in this study align with those observed in previous studies [8,15], both in terms of age and the proportion of female patients operated on compared to males, given that females have an increased risk of hip fractures [16]. Similarly, the distribution of fracture types and their associated surgeries is consistent with previous publications in similar clinical settings [15].

Hip fractures account for approximately 20% of osteoporotic fractures; however, they represent a serious public health problem as they consume the most healthcare resources and cause the highest disability or mortality among those over 50 years old [17]. In our setting, 86.7% of fractures in women and 80.7% in men occur in those over 75 years old [8]. Globally, an increase in the prevalence of hip fractures has been documented, largely attributed to the continuous aging of the population [9].

In this context, aging, even in healthy patients, brings about a physiological decline known as frailty, characterized by increased vulnerability to internal and external stressors, which heightens the likelihood of adverse health outcomes [10,18,19]. From a cardiovascular perspective, there is a dysautonomia marked by an attenuated beta-receptor response, resulting in a reduced ability to maintain cardiac output through increased heart rate. This condition makes elderly patients more dependent on vascular tone and preload to maintain blood pressure levels and, subsequently, more sensitive to hypotensive episodes when subjected to the sympathectomy effects of neuraxial techniques or the vasodilatory effects of most intravenous or inhaled anesthetic agents, as well as hypovolemia frequently associated with hip fractures and their surgical correction [20,21]. Additionally, elderly patients often present with multiple comorbidities across different organ systems and accompanying polypharmacy, which further exacerbates this situation. For this reason, recent clinical guidelines [7] on hip fracture emphasize the importance of close intraoperative hemodynamic control during these interventions, while recommending dosage adjustments of anesthetics tailored to these patients’ specifics.

In this sense, White et al. in two retrospective studies on 11,085 and 1131 patients [2,6], observed a relationship between the dose of subarachnoid anesthesia used and the intraoperative blood pressure levels during hip fracture surgeries, highlighting the need for future studies to explore the use of lower doses of neuraxial anesthesia to maintain normotension.

Investigating this observation, the same research group recently published a retrospective observational study, where, after analyzing 280 patients, they observed that the use of 6.5 mg of 0.5% hyperbaric bupivacaine (1.3 mL) in subarachnoid anesthesia provided an adequate anesthetic duration with minimal changes in blood pressure [22]. Additionally, Mounet et al. conducted a prospective study on 593 patients [23], where they observed the lowest incidence of hypotensive episodes occurred in the group of patients anesthetized under lumbar plexus, quadratus lumborum, and parasacral blocks (22 episodes), compared to patients anesthetized with subarachnoid (23 episodes) or general anesthesia (39 episodes).

Based on these two studies, we decided to use lower doses of subarachnoid local anesthetic (5 mg of 0.5% hyperbaric bupivacaine) combined with performing the PENG and FLCN blocks in the sample of the PENG-CAD trial [14]. The most significant finding of the present study is the statistically significant differences between the groups studied in both the number and duration of hypotensive episodes, favoring the group that used a lower dose of subarachnoid anesthesia supplemented with the PENG block, thus confirming the main proposed hypothesis. These results could be attributable to the reduction in the subarachnoid 0.5% bupivacaine dose (5 mg) by approximately 50–66% compared to that recommended in anesthesiology texts [24] to achieve an adequate block up to the T10 level for hip surgery (10–15 mg), thereby aligning with the goals of dose reduction and adaptation proposed in the latest clinical guidelines for hip fracture.

However, one possible issue with this dose reduction would be failing to achieve a sufficiently high, deep, or prolonged subarachnoid block to carry out the intervention adequately [25]. In the study by Lemoine et al. [26], after analyzing twenty-three studies about subarachnoid anesthesia for lower limb surgery, they found that the Emax for recovery of motor function was 268 min [95% CI (189 to 433 min)] and the D50 was 3.9 mg [95% CI (2.3 to 6.2 mg)]. They concluded that a dose of 7.5 mg of subarachnoid bupivacaine was the most appropriate within the context of low-dose subarachnoid administration, as it allowed for ambulation within 300 min post-surgery in 95% of patients without resulting in insufficient blocks. However, doses of 5 mg or lower presented up to an 8% incidence of insufficient blocks, which the authors deemed unacceptable. Valanne et al. have documented failure rates of 1.9% and 6.2% in patients receiving 4 and 6 mg of subarachnoid bupivacaine, respectively, for knee athroscopy [27], and Janowski et al. found a 4.7% failure rate with a 6 mg dose combined with fentanyl 15 mcg for knee arthroscopy [28]. The hypothesis that emerged from this team, inspired by Mounet et al.’s work [23], is whether the qualities of the PENG and FLCN blocks could not only improve perioperative analgesia with the proven benefits that this entails, but moreover be leveraged to safely reduce subarachnoid anesthesia doses without compromising sufficiently high, dense, and long-lasting intraoperative anesthesia, owing to this technique where PENG blocks nociception in the most densely innervated portion of the hip joint, the anterior capsule, by blocking the sensory articular branches of the femoral, obturator, and accessory obturator nerves¸ added to the blockade of the cutaneous area where the surgical incision is performed in this type of surgeries, provided by the FLCN block. Ultimately, this factor would contribute proportionally to preventing the occurrence and severity of hypotensive episodes. Despite these reported previous results, in our study we observed that of the 108 patients operated on under the combination of 5 mg hiperbaric bupivacaine 0.5% + fentanyl 10 mcg subarachnoid anesthesia and PENG + FLCN blocks, none required supplementary anesthesia during surgery, thus achieving a significant reduction in subarachnoid anesthesia doses without compromising intraoperative anesthesia quality and confirming our hypothesis.

After reaching this conclusion, we set out to compare our study with previous observational and randomized studies on the use of minidose subarachnoid anesthesia in the context of hip surgery. Table 4 provides a visual summary of the relevant findings reported in previous studies, against which we compare the results obtained in our own research. Regarding observational studies, White et al.’s research involving 280 [22] and 11,085 [2] patients, respectively used 6.5 mg and 7.5 mg of 0.5% hyperbaric or isobaric bupivacaine as low-dose subarachnoid anesthesia. In the study by Wood et al. [6], which included 1131 patients, cases were observed where 5 mg or even 4 mg of 0.5% hyperbaric bupivacaine was used, but the need for supplemental intraoperative analgesia was not addressed.

Considering randomized clinical trials, three of them used higher doses of subarachnoid bupivacaine in the low-dose group compared to our study. Minville [29] et al.’s study involving 74 patients employed 7.5 mg of 0.5% isobaric bupivacaine as low-dose subarachnoid anesthesia, which was compared to continuous subarachnoid anesthesia with 2.5 mg boluses of 0.5% bupivacaine every 15 min. The study by Olofson et al. [30], conducted on 50 patients, established 7.5 mg of 0.5% hyperbaric bupivacaine plus 5 mcg of sufentanil as low-dose subarachnoid anesthesia. Martyr et al. [31], in their study of 40 patients, administered 9 mg of 0.5% isobaric bupivacaine with 20 mcg of fentanyl as low-dose subarachnoid anesthesia, which resulted in failed block in 4 patients.

The clinical trial by Errando et al. [32] included 64 patients and administered 3.75 mg of 0.25% hypobaric bupivacaine as low-dose subarachnoid anesthesia. However, they noted that 50 mcg of fentanyl was routinely administered prior to patient mobilization for subarachnoid anesthesia, along with 5–20 mg of ketamine if supplemental analgesia was needed before the start of surgery. This implies the administration of other analgesic drugs in the immediate preoperative period, in addition to subarachnoid anesthesia. Furthermore, they reported that in cases requiring supplemental intraoperative analgesia, ketamine and/or fentanyl were administered, and these patients were excluded from this study, suggesting that the 3.75 mg dose was not always sufficient to achieve adequate anesthesia throughout the entire intraoperative period.

In the study by Ben-David et al. involving 20 patients [33], 4 mg of 0.5% isobaric bupivacaine was used as low-dose subarachnoid anesthesia. However, they also administered fentanyl at a dose of 1 mcg/kg as premedication prior to patient mobilization for subarachnoid anesthesia, as well as up to two intraoperative boluses of 1 mcg/kg fentanyl if supplemental analgesia was required. Thus, in this study as well, the subarachnoid anesthesia dose did not consistently provide sufficient anesthetic depth throughout the surgery.

Kahloul et al. conducted a study on 108 patients [34], establishing a dose of 5 mg of 0.5% hypobaric bupivacaine and 25 mcg of fentanyl as low-dose subarachnoid anesthesia. Additionally, they performed an iliofascial block with 20 mL of 1% lidocaine with adrenaline. Despite this, there were two cases where this subarachnoid anesthesia dose was insufficient during surgery. The meta-analysis led by Messina et al. [35], which included six studies, five of which are mentioned above, found that the average dose of local anesthetic used in these low-dose groups was 6.5 mg.

Therefore, none of the previous studies on low-dose subarachnoid anesthesia in the context of hip surgery had successfully employed such low doses of subarachnoid anesthesia without the need for supplemental intraoperative analgesia, as was achieved in the present study. This constitutes the original contribution of our work to this field of study. In fact, in the only clinical trial where a peripheral nerve block was used in conjunction with low-dose subarachnoid anesthesia, this milestone was neither achieved: in the study by Kahloul et al. [34], despite using the same dose of subarachnoid bupivacaine as in our work (5 mg) in addition to an iliofascial block, there were two cases where this subarachnoid anesthesia dose was insufficient during surgery. This fact further supports our hypothesis in favor of the combination of the PENG and FLCN blocks.

Regarding secondary objectives, no statistically significant differences were observed between groups in terms of vasopressor use, although doses were lower in group B, consistent with the fewer hypotensive episodes in this group. Given that this is a retrospective observational study, no prior sample size calculation was performed to determine the necessary patient sample to achieve statistical significance for these variables. Instead, these results are the outcome of including all patients who underwent hip fracture surgery at our institution in 2022 and met the inclusion criteria. Thus, the observed differences are likely not statistically significant due to an insufficient sample size concerning the use of vasoactive agents.

We find interesting to discuss the difference in the duration of the intervention between groups, which was a factor that surprised us and could, in itself, contribute to the group with blocks experiencing fewer hypotensive episodes than the group without blocks. As well, it could have an impact on the needs of intraoperative supplemental analgesia. Nevertheless, we attribute this different duration of the intervention between groups to a specific detail: the surgical time does not begin with the surgical incision but rather from the patient’s arrival in the operating room for the administration of subarachnoid anesthesia (the PENG and FLCN blocks are performed in a block room prior to the transfer to the operating room). In this regard, we found that patients who underwent the blocks arrived in the operating room with their hip fracture already treated with analgesics, which made the process of placing and administering subarachnoid anesthesia quick and straightforward—a fact supported by previous clinical trials [36,37,38,39,40]. However, patients without the blocks required more time for placement due to the discomfort from the fracture, and the quality of the placement was also inferior, both of which are demonstrated by these clinical trials. Consequently, this extended the time required for administering subarachnoid anesthesia and, in turn, affected the variable “duration of surgery.” Therefore, we do not believe it is likely that this situation led to selection bias, as the strict surgical time from incision to skin closure is probably similar in both groups. However, we acknowledge that the definition of this variable could have been more precise by excluding the time associated with subarachnoid anesthesia from the variable.

### Study Limitations

A great point of debate is the blood pressure limit chosen to define hypotensive episodes. In this context, studies and debates regarding the definition of hypotension have been extensive without reaching an absolute consensus on this concept [41,42,43]. Moreover, it is worth mentioning the additional difficulty of defining this context’s transposition from generality to the individuality of each specific patient. For example, the lower limit of cerebral autoregulation has been observed to range between 40 mmHg and 90 mmHg of MAP [44]. According to the most extensive study to date (11,086 patients) on intraoperative hemodynamic evolution in these interventions [2], the lowest average MAP recorded was 60 mmHg (SD 12 mmHg). Based on this result, we raised the figure chosen to 65 mmHg of MAP as the definition of hypotension and the threshold before starting vasopressors, since as noted in White et al.’s study, different surgeries can have a wide range of minimum blood pressures around these established thresholds (according to their study of ±12 mmHg around the mean of 60 mmHg), and it should be considered that elderly patients, given their frailty and comorbidities, require higher blood pressure levels to maintain adequate organ perfusion compared to young and healthy patients [43].

Another limitation of this study lies in the possibility that various vasoactive stimuli may have influenced the observed blood pressure variations, beyond those directly attributable to the administration of subarachnoid bupivacaine. Such stimuli include factors such as immobility, preoperative nerve blocks, fracture type, movement, surgical manipulation and hip relocation, the use of bone cement, the volume of IV fluids administered during the intervention, active warming measures, sedation variables, and the use of alpha- and beta-blockers. It should be noted, however, that previous randomized controlled trials [29,30,31,32,33,34] also failed to account for these confounding variables, with the exception of objective vasopressor administration. An aspect to consider is the fact that our study is a retrospective observational study, and therefore, it is more susceptible to potential confounding factors compared to randomized studies. However, the observed behavior of blood pressure levels in relation to the dose of subarachnoid anesthetic shows a quantitative relationship similar to that observed in larger randomized clinical trials [29,30,31,32,33,34] and larger retrospective studies reviewed [2,6,22]. As a result, the findings of this work appear to have sufficient consistency and legitimacy to be considered.

Another limitation factor that cannot be downgraded is the fact that this is a retrospective observational study, so absolute randomization between the two groups was not possible, which introduces potential selection and observer biases. Additionally, the sample size was not predetermined, because this study is retrospective in nature, and the patients included in the control group were those who underwent hip fracture surgery at our institution, but could not receive nerve blocks, during the course of the PENG-CAD clinical trial, as referenced in citation number [14]. Despite this fact, the sample size collected in this study was sufficient to demonstrate statistically significant differences in the primary variable; therefore, we do not consider it a major limitation.

For all these reasons, we could consider this work as a pilot study that, given its interesting findings regarding the indirect hypotensive episodes reduction provided by PENG and FLCN blocks—owing to the possibility they offer to reduce doses of subarachnoid anesthesia, without causing it to be insufficient and requiring intraoperative analgesic rescue—could justify the conduct of a prospective randomized study to confirm this benefit with a higher level of evidence.

## 5. Conclusions

The reduction in local anesthetic dosage in subarachnoid anesthesia emerges as one of the most effective maneuvers to prevent intraoperative hypotensive episodes in hip fracture surgery for elderly patients. The use of peripheral nerve blocks, such as the PENG and FLCN, can play a role in this scenario, aiming to maintain adequate intraoperative anesthesia depth and duration while enabling even further reductions in the subarachnoid anesthesia dosage.

## Figures and Tables

**Table 1:** Patient demographic and clinical parameters.

	Group B (n = 108)	Group S (n = 76)	*p*
Age, years (median (IQR))	86.5 (16.0)	90.0 (6)	*p* = 0.853
Gender, n (%)			*p* = 0.401
Male	24 (22.22)	21 (27.63)	
Female	84 (77.78)	55 (72.37)	
Duration of surgery, min (mean (SD))	52.63 (18.40)	73.24 (26.12)	*p* = 0.000
Type of fracture, n (%)			
Subcapital	38 (35.19)	24 (31.58)	
Pertrochanteric	67 (62.04)	47 (61.84)	
Subpertrochanteric	1 (0.95)	4 (5.26)	
Others	2 (1.85)	2 (2.63)	
Type of surgery, n (%)			*p* = 0.919
Partial Hip Arthroplasty	38 (35.19)	28 (36.84)	
Endomedullary Nailing	69 (63.89)	46 (60.53)	
Others	1 (0.93)	1 (1.32)	

Group B: case cohort (subarachnoid anesthesia + PENG block), Group S: control cohort (subarachnoid anesthesia).

**Table 2:** Distribution of time and number of episodes of hypotension between groups.

	Study Group	Median (IQR)	*p*
Time of hypotension (min)	Group S	20 (16)	0.000
	Group B	10 (5)	
Episodes of hypotension (n)	Group S	2 (1)	0.000
	Group B	4 (3.25)	

Group B: case cohort (subarachnoid anesthesia + PENG block), Group S: control cohort (subarachnoid anesthesia).

**Table 3:** Distribution of vasoconstrictor consumption between groups.

Vasoconstrictor	Study Group	Median (IQR)	*p*
Efedrine (mg)	Group S	12 (18)	0.463
	Group B	9 (5)	
Fenilefrine (mg)	Group S	0.25 (0.20)	0.056
	Group B	0.20 (0.23)	

Group B: case cohort (subarachnoid anesthesia + PENG block), Group S: control cohort (subarachnoid anesthesia).

**Table 4:** Summary of Previous Studies on Low-Dose Spinal Anesthesia in Hip Fracture Surgery.

Study	Type of Study	Bupivacaine Dose Used	Need for Supplementary Analgesia
White	Retrospective	6.5–7.5 mg 0.5%, hiper/isobaric	
Wood	Retrospective	4–5 mg 0.5%, hiperbaric	Not stated
Minville	Prospective	7.5 mg 0.5%, isobaric	
Olofson	Prospective	7.5 mg 0.5%, isobaric	
Martyr	Prospective	9 mg 0.5%, isobaric	
Errando	Prospective	3.75 mg 0.25%, hypobaric	Ketamine and fentanyl, PO + IO
Ben-David	Prospective	4 mg 0.5%, isobaric	Fentanyl, PO + IO
Kahloul	Prospective	5 mg 0.5% hypobaric + IFB	2 cases of insufficient SA

The need for supplemental analgesia is reported only in those studies that employed doses lower than, or comparable to, those used in our study. PO: preoperative, IO: intraoperative, SA: subarachnoid anesthesia.

## Data Availability

The original contributions presented in this study are included in this article. Further inquiries can be directed to the corresponding author(s).
